# Arterial Thrombosis in Acute Respiratory Infections: An Underestimated but Clinically Relevant Problem

**DOI:** 10.3390/jcm13196007

**Published:** 2024-10-09

**Authors:** Anastasiya S. Babkina, Mikhail V. Pisarev, Andrey V. Grechko, Arkady M. Golubev

**Affiliations:** Federal Research and Clinical Center of Intensive Care Medicine and Rehabilitology, Moscow 107031, Russia; pisarev@gmail.com (M.V.P.); avg-2007@ya.ru (A.V.G.); arkadygolubev@mail.ru (A.M.G.)

**Keywords:** arterial thrombosis, COVID-19, pneumonia, ARDS, respiratory infections

## Abstract

During the COVID-19 pandemic, there was increased interest in the issue of thrombotic complications of acute respiratory infections. Clinical reports and pathological studies have revealed that thrombus formation in COVID-19 may involve the venous and arterial vasculature. As thrombotic complications of infectious respiratory diseases are increasingly considered in the context of COVID-19, the fact that thrombosis in lung diseases of viral and bacterial etiology was described long before the pandemic is overlooked. Pre-pandemic studies show that bacterial and viral respiratory infections are associated with an increased risk of thrombotic complications such as myocardial infarction, ischemic stroke, pulmonary embolism, and other critical illnesses caused by arterial and venous thrombosis. This narrative review article aims to summarize the current evidence regarding thrombotic complications and their pathogenesis in acute lower respiratory tract infections.

## 1. Introduction

The COVID-19 pandemic has highlighted the thrombotic complications associated with respiratory infections. Since the beginning of the pandemic, attention has been focused on the problem of venous thromboembolism (VTE) in COVID-19 [1,2,3], which has influenced the treatment strategy and preventive measures, including the ultrasonography of the lower extremity veins and prescription of low-molecular-weight heparins to all hospitalized patients without contraindications to anticoagulants [4,5,6]. However, clinical reports and pathological studies indicate that thrombus formation in COVID-19 may spread beyond the venous system and involve the arterial vasculature, particularly as the in situ thrombosis of pulmonary artery branches, despite the use of low-molecular-weight heparins [7,8,9,10,11]. It is important to note that arterial thrombosis as a complication of respiratory infections is not limited to COVID-19 patients. Pre-pandemic studies show that bacterial and viral respiratory infections are associated with an increased risk of thrombotic complications such as myocardial infarction, ischemic stroke, pulmonary embolism, and other critical illnesses caused by arterial and venous thrombosis [12,13,14,15]. It is noteworthy that severe pulmonary infections can lead to acute respiratory distress syndrome (ARDS). Coagulopathy and pulmonary vascular thrombosis have been noted since the beginning of pathologic descriptions of ARDS of various etiologies [16]. As a result, several questions have been raised about the clinical manifestations and pathogenesis of thrombotic complications in acute lower respiratory tract infections.

This narrative review article aims to summarize the current evidence regarding thrombotic complications and their pathogenesis in acute lower respiratory tract infections.

## 2. Materials and Methods

We searched the PubMed database for original research articles, clinical reports, review articles, editorials, commentaries, and short communications published through 25 July 2024. The search was conducted using the set of keywords detailed in Table 1. Articles were selected based on the relevance of the title and abstract to the topic of this review. Additional studies that were not captured through the primary database search were included after manually examining the reference lists of the selected articles. Information considered more relevant was selected. With the aim of discussing the topic more holistically, we decided to forego the systematic review in favor of a narrative review.

## 3. Results

During the COVID-19 pandemic, there was increased interest in the issue of thrombosis in lower respiratory tract infections, as evidenced by a PubMed search for relevant studies. A search for “(respiratory infection) AND (thrombosis)” yields over 7.5 thousand studies. However, the vast majority of sources are dedicated to thrombosis in COVID-19, while the number of studies not related to COVID-19 “((respiratory infection) AND (thrombosis)) NOT (COVID-19)” was 2477 at the time of the search. The final number of papers selected for this manuscript was 223, with the consensus of the authors. As thrombotic complications of infectious respiratory diseases are increasingly considered in the context of COVID-19, the fact that thrombosis in lung diseases of viral and bacterial etiology was described long before the pandemic is overlooked.

### 3.1. Clinical Aspects of the Problem

#### 3.1.1. Myocardial Infarction and Ischemic Stroke: Potential Association with Acute Lower Respiratory Tract Infections

Understanding the relationship between myocardial infarction (MI), the leading cause of death globally, and lower respiratory tract infections, the leading cause of death among infectious diseases, is critical for global public health.

There have been reports of type 1 MI associated with coronary atherothrombosis and type 2 MI, which are usually caused by an imbalance between oxygen delivery/blood supply and myocardial demand due to hypoxemia, fever, and tachycardia in patients with infectious lung diseases [17]. Collins et al. first reported the association between seasonal influenza activity and cardiovascular mortality, which served as the basis for developing the hypothesis that respiratory infections are associated with an increased risk of cardiovascular complications [18]. Numerous studies, systematic reviews, and meta-analyses have since confirmed that respiratory viral infections (including influenza and SARS-CoV-2) and bacterial pneumonia play a role in the development of myocardial infarction and ischemic stroke [15,17]. In 1998, Meier et al. reported the results of an analysis of the General Practice Research Database (GPRD), which showed a significant association between myocardial infarction and respiratory infection in the previous month [19]. Using this database, Clayton et al. found that minor symptoms such as cough, runny nose, nasal congestion, or sore throat were not associated with MI, whereas symptoms of lower respiratory tract infections (pleural pain and fever) were strongly linked to MI [20]. A study using another general practice database, IMS Disease Analyzer (Mediplus), confirmed an increased risk of MI and stroke within 7 days of infection and found that the association with MI persisted regardless of the baseline cardiovascular risk [21]. Individuals with influenza and COVID-19 have been found to have an increased risk of myocardial infarction both during the acute phase of illness and after viral clearance, suggesting a long-term effect on the host’s coagulation and immune systems that begins with infection but may persist long after viral elimination [22]. It has been established that the risk of cardiovascular complications following acute respiratory infections is 2–6 times higher and may persist for up to a month, with the highest risk occurring within the first week of infection [13,14,23].

Myocardial infarction has been reported in 5–7% of patients hospitalized for pneumococcal pneumonia [24,25], with the highest risk occurring during the first days after infection. A prospective observational study conducted by the FADOI-ICECAP research group found an increased risk of cardiovascular complications in individuals with community-acquired pneumonia [26]. In addition, the presence of cardiovascular complications in pneumonia caused by *Streptococcus pneumoniae* is strongly influenced by the serotype of the pathogen, with serotypes 3 and 9n being most frequently associated with such complications [27]. An association between myocardial infarction and infections caused by Mycoplasma pneumonia and Chlamydia pneumoniae has been reported [28,29].

Cardiac complications of COVID-19, including myocardial infarction, were reported during the pandemic [30,31,32]. A meta-analysis by Zuin et al. based on data from 2765 publications found that COVID-19-recovered patients had a higher risk of myocardial infarction than those who did not experience a COVID-19 infection [33]. However, when the complication rates for COVID-19 (89,530 patients) and influenza (45,819 patients) were compared, myocardial infarction and atrial fibrillation were less common in COVID-19 patients than in influenza patients [34]. Ward et al. discovered a similar risk of myocardial infarction in COVID-19 patients and influenza patients [35].

The hypothesis of an association between acute infectious diseases and stroke has existed since the late 19th century, based on the pioneering works of P. Marie and S. Freud on stroke in children [36]. Several subsequent studies have confirmed the association of stroke in children with previous infections, including respiratory infections [37].

Zurrú et al. found a significant association between recent respiratory infections and atherothrombotic stroke [38]. Patients with pneumococcal pneumonia have been shown to have a higher risk of stroke than the general population [39]. *Mycoplasma pneumoniae* was found to be independently associated with the risk of ischemic stroke [40].

According to Boehme et al., acute respiratory infections increase the short-term risk of stroke, especially in people under the age of 45, and can thus be considered a trigger for stroke [41].

According to Luo et al., the cumulative incidence of ischemic stroke during COVID-19 was 2% [42]. We found similar results when analyzing COVID-19 deaths based on autopsy reports [11,43]. Beach et al. found that the incidence of ischemic and hemorrhagic stroke in patients with COVID-19 pneumonia was similar to the incidence of an acute cerebrovascular accident (ACVA) in patients with non-COVID-19 pneumonia [44]. When comparing the incidence of stroke in COVID-19 and influenza, the findings are contradictory. Merkler et al. reported that approximately 1.5% of COVID-19 patients who presented to the emergency department or were hospitalized had an ischemic stroke, which was 7.5 times higher than the rate in patients with influenza [45]. Ward et al., on the other hand, found that patients with COVID-19 and influenza had the same risk of ischemic stroke [35].

#### 3.1.2. Pulmonary Embolism and Pulmonary Artery Thrombosis In Situ as Complications of Respiratory Infections

The combination of pulmonary embolism (PE) and pneumonia is a major concern in critical care medicine.

In 2006, Smeeth et al. published a study showing that the risk of deep vein thrombosis (DVT) after respiratory infection increases in the first two weeks and can persist for up to six months [46]. According to Clayton et al., there was an increased risk of PE within three months of infection, which may persist for up to one year [47]. Ribeiro et al. found that patients with a history of pneumonia were five times more likely to develop venous thrombosis than study participants who had no history of pneumonia. At the same time, the risk of pulmonary embolism exceeded that of DVT alone [48]. According to an analysis of several reports of VTE in COVID-19, the prevalence of PE was higher than that of DVT [49]. The findings of a meta-analysis by Birocchi et al. highlight the disproportionate prevalence of PE among all thromboembolic complications in COVID-19 patients, most likely due to pulmonary artery thrombosis in situ rather than peripheral venous thrombus embolism [50]. According to Poissy et al., the incidence of PE in patients admitted to the ICU with COVID-19 was twice that of patients admitted to the ICU during the same period in 2019 (pre-pandemic). However, the authors point out that the low incidence of coexisting deep vein thrombosis in COVID-19 patients could indicate that they were more likely to have pulmonary artery thrombosis than embolism [51].

Most clinical studies assume that a thrombus in the branches of the pulmonary artery is caused by thromboembolism from the deep veins of the lower extremities. However, there is increasing evidence that de novo thrombosis in the pulmonary arteries can occur even in the absence of DVT [52]. The possibility of in situ thrombus formation in the pulmonary arteries without deep vein thrombosis (DVT) has been demonstrated in studies conducted both during and before the COVID-19 pandemic [52,53,54,55]. Whole-body magnetic resonance imaging for thrombus visualization confirmed the possibility of pulmonary artery thrombosis in the absence of DVT [56]. Based on a detailed analysis of computed tomography pulmonary angiograms (CTPAs) in patients with severe COVID-19, Desborough et al. suggest that segmental and subsegmental changes on CTPAs represent in situ thrombi rather than pulmonary emboli [57]. It is important to consider that other papers looking at VTE in COVID-19 may have overestimated rates of VTE, including thrombosis in situ as PE.

Long before the COVID-19 pandemic, pulmonary arterial thrombosis in ARDS patients was described in pathological studies and clinical research using bedside pulmonary artery balloon occlusion angiography [58]. During the 2003 viral pneumonia epidemic caused by the SARS virus of the coronavirus family, primary pulmonary artery thrombosis that was not associated with DVT was also reported [59]. Pulmonary artery branch thrombosis has been described based on autopsies of patients with fatal H1N1 influenza [60].

#### 3.1.3. Intracardiac Thrombosis in Patients with Respiratory Infections

Thrombosis of the right atrium or ventricle can be a source of pulmonary embolism. For example, in our previous study presenting the spectrum of thrombotic complications in patients who died from COVID-19, the source of thromboembolism was mostly lower extremity veins, but in two of the sixteen cases, the thrombi originated from the right atrium [11]. Several case reports of COVID-19-related intracardiac and aortic thrombosis have been published [61,62,63,64,65,66,67].

Cases of intracardiac thrombosis have been reported not only in COVID-19 but also in *Mycoplasma pneumonia* infection [68], aspergillosis [69], and influenza-associated pneumonia [70]. A case of large thrombus formation in the right ventricle has been reported in a patient with alcohol-induced dilated cardiomyopathy and signs of bacterial lower respiratory tract infections [71].

#### 3.1.4. Mesenteric Thrombosis in Respiratory Infectious Diseases

Acute mesenteric ischemia as a complication of infectious lung disease is mainly considered in the context of COVID-19 [72,73]. There have been reports of both venous and arterial mesenteric thrombosis in COVID-19 [73,74]. Navarro-Martínez et al. reported mesenteric ischemia as a manifestation of COVID-19 [75]. Sureshkumar et al. reported a case of acute mesenteric ischemia caused by superior mesenteric artery thrombosis in COVID-19, with a fatal outcome despite prophylactic enoxaparin [76].

Mesenteric arterial thrombosis with subsequent mesenteric ischemia has also been reported in influenza B [77], pneumonia caused by *Mycoplasma pneumoniae* [78], and *Klebsiella pneumoniae* [79].

#### 3.1.5. Acute Lower Extremity Arterial Thrombosis in Severe Respiratory Infections

Published clinical cases, systematic reviews, and studies suggest that arterial thrombosis of the lower extremities is not uncommon in COVID-19 and is associated with high mortality, amputation rates, and ineffective interventions [80,81,82,83,84]. However, reports of lower extremity gangrene due to thrombosis as a complication of infectious lung disease are not limited to the COVID-19 period. Rinaldo et al. described a case of a patient with ARDS due to *Escherichia coli* pneumonia who developed massive gangrene of both lower extremities requiring a bilateral above-knee amputation [85]. Histologically, the small vessels of the amputated limbs showed thrombi with fibrin and leukocytes. Routine coagulation laboratory tests revealed no clinical signs of disseminated intravascular coagulation (DIC) in this patient.

Thrombosis of the popliteal and femoral arteries has been described in pneumonia caused by *Mycoplasma pneumoniae* [86,87]. Two cases of varicella pneumonia in adults complicated by severe ischemia of the lower extremities caused by thrombosis of the femoral and tibial artery without obvious signs of DIC or fulminant purpura have been reported [88].

#### 3.1.6. Diagnostic Aspects of Thrombotic Complications of Acute Lower Respiratory Tract Infections

Numerous studies, clinical reports, reviews, and meta-analyses indicate that acute respiratory tract infections are associated with an increased risk of thrombotic complications, including various forms of pulmonary and extrapulmonary thrombosis. Table 2 summarizes thrombotic complications in pulmonary infections of the various etiologies described in the cited sources.

Extrapulmonary arterial thrombosis can manifest as myocardial infarction, stroke, acute lower limb ischemia, and mesenteric ischemia, each with distinct signs that can be detected by clinical assessment and imaging techniques. However, timely diagnosis requires clinical vigilance and the physician’s awareness of the increased risk of thrombotic events in severe acute lower respiratory tract infections, particularly in patients with cardiovascular comorbidities.

Identifying thrombotic events in the lungs during lower respiratory tract infections presents several challenges: (1) PE can be a complication of pneumonia, as well as be the cause of postinfarction pneumonia, making differential diagnosis difficult [89]; (2) symptoms and signs of pulmonary thrombosis often occur in severe respiratory infections characterized by pre-existing respiratory failure, making clinical diagnosis difficult; (3) the lack of clear criteria for differentiating PE from in situ pulmonary artery thrombosis may lead to the misclassification of in situ thrombosis as PE. Thus, although discussed in a radiologic review article from 1951 [90], in situ pulmonary artery thrombosis has received little attention from the medical community [91].

The diagnosis of in situ thrombosis, which primarily affects the distal pulmonary arteries, requires techniques that can visualize segmental and subsegmental pulmonary vessels. Currently, CT pulmonary angiography (CTPA) is the most widely used and validated imaging modality for the diagnosis of pulmonary embolism (PE) and has demonstrated efficacy in identifying occlusions in segmental and subsegmental branches [92].

Experience gained during the pandemic has led to the establishment of key indications for CTPA in lower respiratory tract infections, including symptoms of PE, high oxygen requirements despite minimal lung damage, unexplained severe respiratory failure, coagulation abnormalities, hemodynamic instability, and right atrial and/or ventricular enlargement [93]. In a case study of Chlamydia psittaci pneumonia, Fang et al. recommended considering pulmonary artery thrombosis and angiography if dyspnea, chest pain, and elevated D-dimer levels persist after targeted antibiotic treatment [94]. This recommendation may also apply to other severe respiratory infections.

However, the evidence for using D-dimer levels to determine CTPA indications remains inconsistent. The clinical use of D-dimer testing is complicated by its high sensitivity but low specificity [93]. Elevated D-dimer levels can occur in several conditions, including cancer, atrial fibrillation, acute coronary syndrome, stroke, major upper gastrointestinal bleeding, infection, disseminated intravascular coagulation, and end-stage renal disease, limiting its usefulness in critically ill patients [93,95]. When studying thrombosis in the context of infection, it is important to recognize that D-dimer is a nonspecific acute phase reactant [96]. Consequently, elevated plasma D-dimer levels in patients with pneumonia may be associated with an enhanced inflammatory response [97]. Crowther et al. demonstrated that D-dimer levels may not be a reliable prognostic or diagnostic indicator of DVT in critically ill patients [98]. A case of acute pulmonary embolism diagnosed despite a negative D-dimer test in the blood serum was described [97]. Therefore, despite its reported high sensitivity, D-dimer levels should not be considered a primary indication for CTPA in suspected pulmonary thrombosis or thromboembolism [93].

The clinical signs of COVID-19-associated thrombotic pulmonary artery occlusion seem to be distinct from those of PE in patients without COVID-19 [99,100]. Thus, thrombi in pulmonary artery branches in COVID-19 patients are primarily found in the most affected region of the lung. Pulmonary artery thrombosis is usually peripheral and is rarely associated with signs of right heart overload [99,101]. A study comparing the CT features of in situ pulmonary artery thrombosis in Hughes–Stovin syndrome (HSS) with those of PE showed that thromboemboli generally appear mobile and loosely adherent, whereas the thrombus observed in HSS is tightly adherent to the vessel wall [102]. Similarly, a tight adherence of thrombi to the vessel wall has been observed in postmortem examinations of COVID-19 patients [11].

A comprehensive approach combining CT venography and pulmonary angiography can also be used to differentiate between pulmonary embolism and in situ thrombosis. This single test not only identifies pulmonary vein occlusion, but also pinpoints the source of the embolus in pulmonary embolism. It surpasses lower-extremity ultrasound testing by enabling the evaluation of deep veins in the abdomen and pelvis [103].

CTPA has become the leading imaging modality, almost replacing ventilation–perfusion lung scintigraphy (VQ scanning) [104]. However, certain clinical scenarios favor scintigraphy over VQ scanning, such as impaired renal function, contrast allergy, or patients’ inability to fit into a CT scanner [105]. Scintigraphy ((SPECT) V/Q imaging) can serve as a complete alternative to CTPA with similar sensitivity and specificity [106,107,108,109]. Tessonnier et al. demonstrated the superiority of scintigraphy over CT angiography in a specific case [110]. In a patient with hyperhomocysteinemia and pulmonary arterial hypertension, pulmonary scintigraphy revealed multiple distal pulmonary artery thromboses, whereas the multidetector CT pulmonary angiography showed normal results.

This method has significant drawbacks for patients with respiratory infections, including potential aerosol escape and the airborne transmission of pathogens during the ventilation phase of VQ, as well as increased coughing episodes after the inhalation of radiopharmaceuticals [93].

Techniques for detecting thrombosis continue to improve, as evidenced by encouraging results from experimental research. De Bruyn et al. proposed the use of radiolabeled TNK-tPA with biochemically inhibited plasminogen activity for the rapid scintigraphic identification of pulmonary and ventricular thrombi [111]. Lim et al. developed a novel recombinant fluoroprobe targeting activated platelets for the in vivo detection of arterial thrombosis and pulmonary embolism using three-dimensional fluorescence emission computed tomography (FLECT) technology [112].

### 3.2. Pathophysiological Aspects of the Problem

The COVID-19 pandemic demonstrated that prophylaxis with LMWH does not completely eliminate the risk of thrombotic complications [93,96]. As arterial thrombi are formed under high shear conditions and are VWF-platelet rich, it can be hypothesized that prescribing LMWH may not be the most efficacious method for preventing arterial thrombotic events. The large number of reports on the prevalence of arterial thrombosis confirms the need to improve thrombotic prevention methods, taking into account the differences in the mechanisms of venous thrombus formation and arterial thrombosis in situ associated with acute infectious respiratory disease.

**Figure 1:**
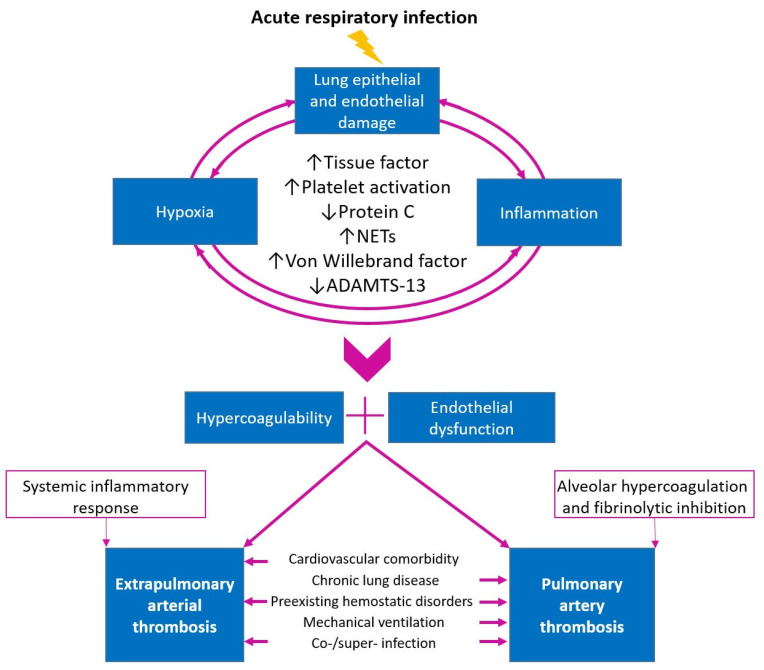
Key components of the pathogenesis of arterial thrombosis in acute respiratory infections.

Inflammation, hypoxia, and damage to alveolocytes and endothelial cells are components of the pathogenesis of infectious diseases of the lower respiratory tract. These factors are mutually aggravating and lead to hypercoagulation and endothelial injury, providing conditions for thrombus formation (Figure 1).

One possible mechanism for thrombotic complications in acute infection is the activation of the hemostasis system [113]. The procoagulant state has long been recognized as a critical component of the pathophysiology of ARDS [114,115]. Hypercoagulability in COVID-19 was reported in the earliest studies [116,117,118]. It was noted that COVID-19 ARDS patients have a higher coagulation potential, while DIC scores is higher in non-COVID-19 ARDS patients [119]. Spiezia et al. showed that patients with COVID-19 and patients with viral and bacterial pneumonia without COVID-19 had significantly hypercoagulable thromboelastometry profiles vs healthy individuals, characterized by a significantly shorter clot formation time and significantly increased maximum clot firmness. However, in patients with COVID-19, the prothrombotic state was more marked. The authors consider the increase in clot firmness in the FIBTEM assay as a pathogenetic factor of pulmonary vascular thrombosis and fibrin deposition in the alveolar and interstitial spaces of the lungs [120]. The determination of the characteristics of coagulation profiles in patients with pneumonia of various etiologies using viscoelastic tests can help in identifying the mechanisms of coagulopathy in various infectious lung diseases.

Endothelial damage is critical in the pathogenesis of respiratory infections. The pulmonary endothelium has antithrombotic properties. The surface receptor CD39, prostaglandin PGI2, and nitric oxide (NO) prevent platelet and leukocyte activation. NO reduces leukocyte adhesion to the endothelium. The endothelial surface also normally contains tissue factor pathway inhibitor (TFPI), which inhibits coagulation initiation, and glycosaminoglycans, which block the binding of prothrombotic factors. Endothelial thrombomodulin activates the potent antithrombotic protein C pathway [121]. However, the endothelium responds rapidly to injury and inflammation by promoting thrombosis and allowing the targeted transmigration of inflammatory cells, such as neutrophils, into the alveolar airspace. The production of the von Willebrand factor plays an important role in thrombus formation, while the surface expression of P-selectin plays a key role in binding neutrophil transmigration [122].

Several studies have demonstrated endothelial cell damage in respiratory viral infections and bacterial pneumonia [123,124,125,126]. Patients with COVID-19-induced ARDS have higher serum levels of endothelial damage markers such as angiopoietin-2, intercellular adhesion molecule-1, vascular cell adhesion molecule-1, P-selectin, and E-selectin than patients with other types of ARDS [127]. Endothelial damage in COVID-19 is confirmed by morphological changes in pulmonary arteries and arterioles, which include endothelial cell loss, vacuolization of their cytoplasm, and degeneration [121]. The same morphological changes in the endothelium of pulmonary vessels are also detected in ARDS of non-COVID etiology [128].

Since the COVID-19 pandemic, the previously used terms “thromboinflammation” and “immunothrombosis” have become more popular and widespread. The term “thromboinflammation” first appeared in the early 1990s in experimental studies on models of cerebral air embolism. Data from ultrastructural and functional studies demonstrated an interaction between air bubbles, blood elements (platelets, fibrinogen, and leukocytes), and the endothelium, resulting in local fibrin deposition and the adhesion of platelets and leukocytes to both bubbles and the air-damaged endothelium. Based on these findings, it was suggested that brain damage from arterial air embolism was caused by local secondary reactions of the air-damaged endothelium rather than the temporary occlusion of the arteries. Thus, to describe the process of thrombosis with a strong cellular response, the authors used the concept of a “thromboinflammatory” response [129].

**Figure 2:**
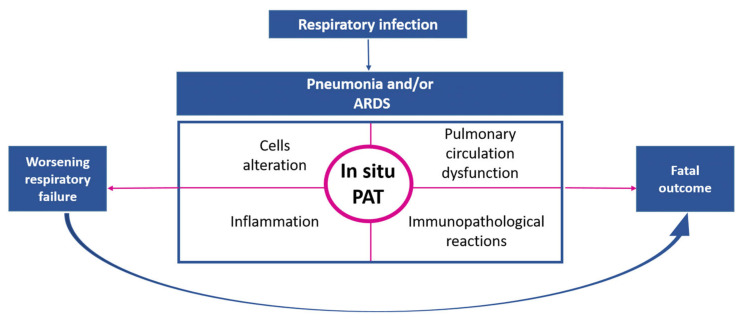
In situ pulmonary artery thrombosis (PAT) in the pathogenesis of acute lower respiratory tract infections.

Later, with the emergence of a large body of evidence for the involvement of the immune system, particularly complement system proteins, in the process of thrombosis [130], the rationale for the introduction of a term covering the interaction between the immune system and hemostasis appeared. Thus, the term “immunothrombosis” was introduced in 2013 [131]. However, the interaction between the hemostasis system and innate immunity is an essential component of thrombosis and has been studied long before the term “immunothrombosis” was coined [132,133,134,135]. These terms are mostly used in studies dedicated to the pathogenesis of pulmonary artery thrombosis in ARDS. The pathogenesis of pulmonary artery thrombosis in infectious lung diseases is a complex interplay of pathological processes, including cell alteration, inflammation, immunopathological reactions, and circulatory dysfunction. Extensive in situ pulmonary thrombosis may exacerbate respiratory failure and lead to death (Figure 2). Therefore, the development of strategies for early diagnosis and pathogenetically based treatment is critical to reduce the risk of adverse outcomes in patients with lower respiratory tract infections.

#### 3.2.1. Role of Tissue Factor in Activation of the Coagulation System during Lung Infection

Based on data confirming tissue factor (TF) production by alveolar macrophages and alveolar epithelial cells during inflammation, several studies have focused on the role of TF in the activation of the coagulation system in respiratory infectious diseases [136,137]. There is evidence that TNF-α and IL-1β control TF production during pulmonary infection [138]. TF released from the alveolar epithelium is thought to contribute to intra-alveolar fibrin deposition in combination with a disbalance of alveolar hemostasis in ARDS [139]. The inhalation of lipopolysaccharide-activated blood coagulation [140,141] results in an increase in the number of TF microparticles [142].

The concept of TF association with thrombotic complications of severe respiratory infections was extended to COVID-19. High TF expression in the lung is thought to promote fibrin formation and platelet activation during COVID-19 infection [143]. Prior to the COVID-19 pandemic, increased TF expression was found to be associated with thrombosis in patients infected with other pathogenic coronaviruses and the influenza A virus [144]. TF has been found in bronchoalveolar lavage from patients with bacterial pneumonia [145].

Many studies on the role of TF in pulmonary infections have led to the identification of TF as a possible therapeutic target. In pneumonia models, inhibiting TF activity prevented the local activation of blood coagulation and reduced intra-alveolar fibrin deposition [146]. The reduction in TF expression via nuclear factor kappa B (NF-κB) inhibitors promotes the attenuation of alveolar hypercoagulation and fibrinolysis inhibition in experimental models of ARDS [147,148]. In pneumonia caused by intranasal *S. pneumoniae* inoculation, the subcutaneous injection of a recombinant nematode anticoagulant protein (rNAPc2) inhibited the circulating TF ligand TF-FVIIa, lowering the procoagulant response in the lung [145]. However, TF on lung epithelial cells has been shown to support pulmonary hemostasis after influenza A (H1N1) virus infection, and inhibiting the TF-dependent activation of blood coagulation increased alveolar hemorrhage [149].

#### 3.2.2. Thrombin and Coagulation Factor FXIIa in the Regulation of In Situ Thrombus Structure

Dense, fibrin-rich arterial thrombi found in situ during severe respiratory infection with ARDS indicate high thrombin activity [121]. One important function of thrombin is to activate platelets via PAR1 and PAR4. Thrombin activates endothelial cells via PAR-1, increasing the release of von Willebrand factor (VWF) from Weibel–Palade bodies. Thrombin also stimulates the expression of P-selectin on the endothelial surface, promoting neutrophil recruitment and activation [121]. Elevated thrombin activity is observed in ARDS of various etiologies, including COVID-19 [128,150]. Based on experimental data, Okamoto et al. showed that recombinant antithrombin (rhAT) attenuates ARDS in endotoxemia [151]. However, in the study by Kipnis et al., intravenous rhAT administration led to further permeability disorders in Pseudomonas aeruginosa-induced acute lung injury [152]. In animal models of bacterial pulmonary infection and lung injury, nebulized antithrombin was able to reduce pulmonary coagulation without altering systemic coagulation and no bleeding [153,154].

High thrombin generation potential and fibrinogen levels facilitate the formation of stable clots composed of a dense network of thin fibrin filaments [155]. Activated Hageman factor (FXIIa) has been shown to regulate clot density independently of thrombin generation [156,157]. Although FXIIa has been shown to convert FXI to FXIa, thereby promoting thrombin generation, the mechanism of its direct effect on fibrin clot architecture remains unknown. Elevated fibrinogen levels have been associated with accelerated FXIIa generation in COVID-19, which may contribute to compact fibrin clot architecture, as suggested by Wygrecka et al. [155]. High levels of FXIIa in bronchoalveolar lavage and their prognostic value have been reported in ARDS of non-COVID etiology [158]. Several studies have shown that targeting FXIIa with 3F7 appears to be a promising approach to treat thrombosis with a minimal risk of bleeding [159,160].

#### 3.2.3. Protein C Dysregulation as a Potential Mechanism of Thrombosis in Respiratory Infections

TF-induced thrombin generation is poorly controlled by physiological anticoagulant mechanisms in the lung [128]. Thrombin interacts with thrombomodulin (TM) to activate protein C, which binds to the endothelial cell protein C receptor (EPCR) [161,162]. Activated protein C (APC) is an endogenous anticoagulant that inhibits factors Va and VIIIa while suppressing cytokine production. In addition, APC stimulates fibrinolysis by suppressing plasminogen activator inhibitor-1 (PAI-1). Ware et al. showed that in patients with ARDS, plasma APC levels were decreased and PAI-1 levels were increased, compared with controls with cardiogenic pulmonary edema [163]. Low APC and high PAI-1 levels were linked to increased mortality in ARDS patients [58]. Another possible mechanism for pneumonia-related thrombosis is impaired protein C production and/or activation [164]. The relative deficiency of the protein C system could be attributed to increased protein C consumption and degradation by neutrophil elastase [165]. Studies demonstrating high levels of soluble thrombomodulin in lung inflammation suggest that thrombomodulin release from the cell surface contributes to protein C deficiency [166,167].

It should be noted that although bronchoalveolar fluid contains some activated protein C (APC), the lung has a very limited ability to produce protein C [168]. Therefore, impaired protein C activation could be attributed to liver cell damage caused by respiratory failure in pneumonia associated with tissue hypoxia [169].

An experimental study showed that mice with heterozygous protein C deficiency had worse outcomes, increased fibrin deposition in the lungs, kidneys, liver, and higher levels of tumor necrosis factor-alpha (TNF-α), interleukin-6 (IL-6), and IL-1β after an intraperitoneal injection of Escherichia coli endotoxin [170]. In a model of Gram-negative pneumosepsis caused by Burkholderia pseudomallei, APC inhibition increased coagulation activation [11,171]. It has been shown that protein C activity is also reduced in COVID-19 [172]. However, the results of studies on the efficacy of recombinant APC in ARDS and sepsis are contradictory. Therefore, uncertainties remain regarding the clinical use of rhAPC [173,174].

#### 3.2.4. Platelet Activation in Severe Respiratory Infections as a Component of Thrombosis Pathophysiology

Several studies have found an association between severe respiratory infections and platelet activation. Platelets are known to contribute to inflammatory responses, particularly by modulating leukocyte effector functions [175] and interacting with microorganisms [176] via toll-like receptors [177]. Activated platelets release the chemokine platelet factor 4 (PF4), which is stored in their granules. PF4 binds to polyanions (P) on bacteria, causing conformational changes and exposing neoepitopes that stimulate antibody production against PF4/P. Because PF4 binds to a wide range of bacteria, anti-PF4/P IgG can bind and opsonize multiple bacterial species. Palankar et al. showed that platelets specifically kill *E. coli* opsonized with PF4 and human anti-PF4/P IgG [178]. *Streptococcus pneumoniae* infection has been found to cause significant platelet activation and hyperreactivity [179,180]. Feldman et al. confirmed that pneumolysin independently activates platelets in vitro, causing homotypic aggregation, as well as mediates the heterotypic aggregation of neutrophils and platelets in vitro, suggesting that pneumolysin may be involved in cardiovascular complications of nosocomial pneumonia [181]. Viral infections, such as influenza, also promote platelet influx into the lungs. Influenza has been linked to increased platelet adhesion to the pulmonary endothelium [182,183].

There is some evidence that platelet aggregation changes during viral respiratory infections, which are most commonly caused by rhinoviruses and coronaviruses (40–65% of all cases) [184]. Kreutz et al. examined platelet surface receptor CD41a (glycoprotein IIb/IIIa), CD62P (P-selectin), and platelet reactivity (ADP-induced aggregation) during viral infection and 6 weeks later. Platelet reactivity and P-selectin expression were higher during upper respiratory tract viral infection compared to 6 weeks later and the control group. Thus, data have been obtained that show increased platelet reactivity and activation in respiratory viral infections [185]. However, major limitations of this study were the lack of data on the etiology of the infection, as well as inclusion and exclusion based on symptoms common to viral (inclusion) and bacterial (exclusion) infections.

Higher levels of platelet activation were also found in COVID-19 compared to controls [186]. McMullen et al. performed an immunohistochemical study of autopsy lung material from patients who died of COVID-19, influenza, and bacterial and fungal infections using antibodies against CD61. An increase in CD61 staining was observed in almost all samples compared to control lung tissue. The area of CD61 staining in COVID-19 infection was larger than in influenza, but still comparable to many other infectious diseases. In aspiration pneumonia, Staphylococcus aureus infection, and blastomycosis, the area of CD61 staining was the largest. The authors concluded that platelet deposition is characteristic of many pulmonary infections [183]. Campo et al. showed an association of heightened platelet activation with the severity of illness, myocardial injury, and mortality in COVID-19. This study also showed that platelet activation is not specific for COVID-19 patients and is also observed in patients with respiratory failure of non-COVID etiology [187].

Given the association of platelet activation with infection and the significant role of platelets in arterial thrombus formation [188,189], platelets need to be considered as a therapeutic target. The development of antiplatelet drugs that interfere with the interaction of platelet receptors with von Willebrand factor (VWF) may provide the basis for new therapeutic strategies that will selectively inhibit arterial thrombosis without interfering with normal hemostasis [190].

#### 3.2.5. The Role of Neutrophils in the Pathogenesis of Thrombosis

Several studies have confirmed the role of neutrophils in pulmonary artery thrombosis, most notably one conducted by Porembskaya et al. in rats with normal neutrophil counts and neutropenia. No thrombus was found in the pulmonary arteries of the neutropenic animals. Neutropenia reduced the size of the thrombus in the inferior vena cava and slowed the transition from fresh to mature fibrin with connective tissue within the thrombus [191].

The pathogenetic link between inflammation and thrombosis was confirmed by thrombus structure studies and lung samples from non-survivors of COVID-19 [192]. Quartuccio et al. investigated twenty arterial thrombi and discovered a high concentration of leukocytes, primarily neutrophils, in their structure [193].

NETosis is one of the mechanisms involving neutrophils in thrombosis. It is a multistep cell death program that includes chromatin decondensation and the formation of neutrophil extracellular traps (NETs). NETs cause endothelial cell damage indirectly via NET-associated proteases, defensins, and histones. The interaction of histones with the endothelium may promote thrombosis by activating endothelial cells and releasing von Willebrand factor from Weibel–Palade bodies. NETs are large structures that, like von Willebrand factor (VWF) and fibrin, may help to maintain thrombus stability. In vitro, NETs were found to provide a framework for clots that are resistant to tissue plasminogen activator (tPA)-induced thrombolysis [194]. Neutrophil extracellular traps have also been linked to the development of thrombotic reactions in COVID-19 and ARDS of various etiologies [195,196]. Intervention regarding NETosis in ARDS is one of the key scientific problems. In a recent study, Liu et al. highlighted the intervention targets of PAD4, including an enzyme regulating the NET skeleton protein histone H3 to citrulline histone to form NETs, potentially providing future therapeutic targets [197].

#### 3.2.6. Von Willebrand Factor and ADAMTS-13 in the Pathogenesis of Arterial Thrombosis In Situ

VWF is made and released by endothelial cells, megakaryocytes, and platelets. Endothelial cells produce the majority of VWF in plasma (80–90%) in response to a variety of damaging factors. VWF forms multimers (ULvWF), the size of which is regulated by the VWF-cleaving protease ADAMTS-13. The metalloproteinase ADAMTS-13 cleaves VWF multimers, which is important for maintaining normal hemostasis because VWF’s functional activity is proportional to the size of multimers. The adhesive properties of ULvWF multimer “strings” promote platelet capture and thrombosis [198].

Accumulating evidence suggests a VWF-mediated mechanism of thrombosis in diseases involving an inflammatory response [199]. An increase in plasma VWF associated with a decrease in ADAMTS-13 has been reported in respiratory infections of both viral and bacterial etiology [200]. The imbalance between VWF and ADAMTS-13 has been attributed to a relative deficiency of ADAMTS-13 due to a significant increase in VWF levels [201]. For example, in COVID-19 patients, this imbalance resulted in large VWF multimers, which were linked to a high thrombotic risk [202,203]. The important role of VWF in the development of thrombosis in COVID-19 is confirmed by significantly more intense immunohistochemical (IHC) staining of VWF in the pulmonary vascular endothelium in patients with thrombotic complications than in patients without thrombotic complications [204].

A procoagulant imbalance has also been observed in patients with nosocomial pneumonia; in particular, the VWF/ADAMTS13 ratio was higher in patients with pneumonia than in healthy subjects [200]. *Streptococcus pneumoniae*, the most common pneumonia pathogen, has been shown to induce Weibel–Palade exocytosis and the release of VWF and interleukin 8 from lung endothelial cells [205]. *Staphylococcus aureus* infection has been shown to be associated with increased levels of neutrophil extracellular traps (NETs) and von Willebrand factor (VWF) and decreased ADAMTS13 activity [206].

Given that patients with severe acute respiratory infections may require mechanical ventilation, which can lead to a variety of complications, including thrombosis [207], studies demonstrating an increase in serum VWF in ventilated patients deserve special attention. Interleukin-6, interleukin-8, surfactant protein D, and soluble tumor necrosis factor receptor I/II (sTNFr I/II), as well as ICAM-1 and VWF, have been found to be elevated in patients with acute lung injury, and their levels fluctuate rapidly in response to different ventilation strategies [208]. Ware et al. discovered that plasma VWF levels increased in response to the duration of mechanical ventilation [209]. Yiming et al. investigated the interaction of platelets with endothelial cells during high tidal volume lung ventilation and discovered that platelets transport platelet-binding proteins, including VWF, to the endothelial cell surface during ventilation [210].

Such research paved the way for the investigation of von Willebrand factor as a therapeutic target. Onodera et al. found that regulating von Willebrand factor by ADAMTS13 reduced lipopolysaccharide-induced lung injury in mice [211]. Zhao et al. showed that the chimeric monoclonal antibody MHCSZ-123 against the human von Willebrand factor A3 domain inhibits arterial thrombosis in a Rhesus monkey model [212]. N-acetylcysteine (NAC) has been shown to inhibit platelet thrombus formation via the degradation of plasma VWF multimers [213]. The results of the study by Kim et al. showed the effectiveness of N,N′-Diacetyl-L-cystine (DiNAC) for the lysis of arterial thrombi. Notably, DiNAC did not lyse red clots. The advantages of DiNAC over tPA, ADAMTS-13, abciximab, and N-acetyl cysteine in relation to arterial thrombus lysis were also revealed [214].

#### 3.2.7. The Role of Co/Superinfection in Thrombotic Complications

The mechanisms of thrombus formation discussed in this review have been observed in both bacterial and viral infections. However, a major limitation of most studies of thrombotic complications in viral infections is the lack of conclusive evidence to rule out bacterial superinfection. The high prevalence of early-onset bacterial coinfection was observed among patients with severe COVID-19 [215]. Based on autopsy protocols and morphological studies of the lungs of COVID-19 patients who died, we found an association between thrombotic complications and the histological evidence of bacterial infection of the lung [11]. We have previously found that bacterial pneumonia is associated with an increased intensity of IHC staining for VWF in the endothelium of pulmonary vessels [204]. Coinfection has been identified as a risk factor for thrombosis in critically ill influenza patients [216].

Walters et al. published the first study of the reconstructed co-infection of H1N1 1918 influenza virus and *Streptococcus pneumoniae* (SP), which deserves special attention in the context of discussions of the role of co-infection in thrombosis [217]. Coinfection was found to cause pulmonary artery thrombosis in H1N1 1918 influenza. Co-infected mice had significantly worse disease, shorter survival times, and more severe lung injury, including widespread thrombosis, high tissue factor (F3) expression, and significant neutrophil elastase deposition on endothelial and epithelial cell surfaces. This study discovered a common feature of influenza 1918 and SP virus co-infection in mice and humans: extensive tissue factor expression and the activation of the extrinsic coagulation pathway, which results in widespread pulmonary thrombosis. Narayana Moorthy et al. showed that in dual viral and bacterial infection, compared with monoinfection, more excessive NET generation was observed, which correlated with exaggerated inflammation and damage to the alveolar–capillary barrier [218]. Therefore, the early detection and treatment of bacterial co/super infection may help reduce the inflammatory response and NET formation, which is important for preventing thrombus formation in acute respiratory infections.

#### 3.2.8. Comorbidity and Thrombotic Complications in Acute Respiratory Infections

Some thrombotic complications in acute respiratory infections result from severe comorbidity and the exacerbation of pre-existing conditions caused by the above mechanisms and hypoxia. This is particularly true for complications such as ischemic stroke and myocardial infarction. We have previously shown that patients who died of cerebral infarction during COVID-19 had severe comorbidities, particularly basilar artery atherosclerosis and stenosis, and a history of acute cerebrovascular accidents [43,219].

Data have been collected on the role of various bacterial and viral infections in the development of atherosclerosis and in the destabilization and rupture of atherosclerotic plaques [220]. An association of histopathological features of plaque instability with *Mycoplasma pneumoniae* and *Chlamydia pneumoniae* infection was revealed [221]. Both the direct participation of infectious agents in the pathogenesis of atherothrombosis and their indirect involvement via immunopathological reactions and systemic inflammatory responses are considered [15,220]. Comorbidity is also associated with pulmonary thrombosis. A high risk of pulmonary thrombosis is observed in patients with chronic lung diseases and vasculitic disorders [53].

## 4. Future Perspectives

According to the available literature, the problem of arterial thrombosis in infectious lung diseases existed long before the COVID-19 pandemic. Acute infectious lung diseases promote a prothrombotic state and endothelial injury, increasing the risk of arterial and venous thrombosis. Local (pulmonary) factors are more closely associated with pulmonary artery thrombosis in situ. The high frequency of arterial thrombosis in respiratory infections confirms the need to improve methods for preventing thrombotic complications, taking into account the pathogenesis of arterial and venous thrombosis. The development of clinical guidelines for the differential diagnosis of pulmonary artery thrombosis and pulmonary embolism is required.

**Figure 3:**
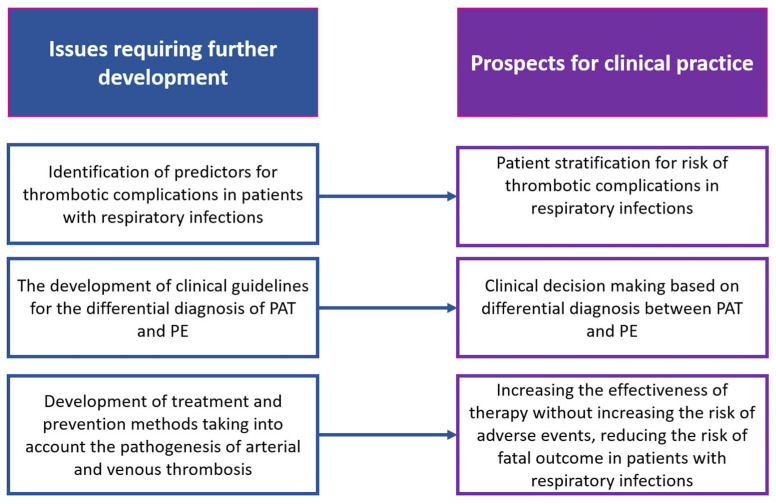
Issues that need to be resolved to improve methods of prevention, diagnosis, and treatment of thrombotic complications of acute lower respiratory tract infections.

Thus, the development of effective strategies for the management of patients with severe pulmonary infectious diseases involves a number of issues to be addressed (Figure 3).

All of the thrombotic mechanisms described in COVID-19 were discovered in pre-pandemic studies of ARDS in viral respiratory infections and bacterial pneumonia. The novelty of the virus, the high morbidity, and the global scope of the problem prompted a large number of studies, most of which were interpreted as COVID-19-specific [222]. However, there is currently no conclusive evidence regarding the specificity or uniqueness of COVID-19 pathogenesis. Clinical and pathological studies have confirmed that severe COVID-19 is most often caused by the development of ARDS. The morphologic substrate of ARDS is characterized by exudative, proliferative, and fibrotic stages detected in autopsy lung material, as well as the thrombosis of pulmonary arteries and arterioles [223]. Therefore, future research should focus on investigating the patterns of different pathogenetic types of ARDS in order to develop more effective clinical guidelines.

## 5. Conclusions

The COVID-19 pandemic has highlighted the critical issue of thrombotic complications of acute respiratory infections, for which no effective preventive measures have yet been developed. Given the high prevalence and pathophysiological background of pulmonary artery thrombosis in situ in lower respiratory tract infections, it is critical to establish guidelines for early diagnosis and treatment. Clinical vigilance for thrombotic complications of acute infectious lung disease, particularly those associated with ARDS, is required in the care of these patients.

## Figures and Tables

**Table 1:** Keywords used for the search strategy of PubMed database.

Search Strategy
(respiratory infection) AND (thrombosis)
((respiratory infection) AND (thrombosis)) NOT (COVID-19)
(pneumonia) AND (thrombosis)
((pneumonia) OR (respiratory infection)) AND (stroke)
((pneumonia) OR (respiratory infection)) AND (myocardial infarction)
((pneumonia) OR (respiratory infection)) AND (pulmonary embolism)
((pneumonia) OR (respiratory infection)) AND (arterial thrombosis in situ)
((pneumonia) OR (respiratory infection)) AND (cardiac thrombosis)
((pneumonia) OR (respiratory infection)) AND (mesenteric thrombosis)
((pneumonia) OR (respiratory infection)) AND (lower extremity thrombosis)
(pulmonary artery thrombosis) AND (diagnostic)
((thrombosis pulmonary) OR (pulmonary embolism)) AND (imaging)
((pneumonia) OR (respiratory infection)) AND (pathogenesis) AND (thrombosis)
(therapeutic target) AND (immunothrombosis)
(thrombosis) OR (immunothrombosis) OR (immunothrombosis) OR ((thromboinflammation) AND (ARDS))

**Table 2:** Arterial thrombotic events in patients with pulmonary infections of various etiologies.

Thrombotic Complication	Etiology of Infectious Diseases
Myocardial infarction	Influenza viruses [14,15,17,18,22,34,35], *Streptococcus pneumoniae* [24,25,27], *Mycoplasma pneumoniae* [28,29], *Chlamydia pneumoniae* [29], SARS-CoV-2 [17,22,30,31,32,33,34,35]
Ischemic stroke	*Streptococcus pneumoniae* [39], *Mycoplasma pneumoniae* [40], Influenza viruses [35,45], SARS-CoV-2 [11,35,42,43,45]
Pulmonary artery thrombosis	SARS-CoV-2 [11,50,51,57], SARS [59], Influenza viruses [60]
Intracardiac thrombosis	SARS-CoV-2 [11,61,62,64], Influenza viruses [70], *Mycoplasma pneumoniae* [68], *Aspergillus* [69]
Mesenteric thrombosis	SARS-CoV-2 [72,73,74,75,76], Influenza B [77], *Mycoplasma pneumoniae* [78], *Klebsiella pneumoniae* [79]
Acute lower extremity arterial thrombosis	SARS-CoV-2 [80,81,82,83,84], varicella pneumonia [88], pneumonia caused by *Escherichia coli* [85], *Mycoplasma pneumoniae* [86,87]
